# Oral care and prevention of pneumonia after withdrawal of nasogastric tube feeding in three elderly patients with psychiatric disorders

**DOI:** 10.1002/ccr3.1294

**Published:** 2017-11-24

**Authors:** Atsushi Hamuro, Minoru Honda, Ryuichi Tanaka

**Affiliations:** ^1^ Department of Psychiatry Yuzuriha Hospital Nagasaki Japan; ^2^ Department of Nursing Yuzuriha Hospital Nagasaki Japan; ^3^ Department of Dentistry Yuzuriha Hospital Nagasaki Japan

**Keywords:** Elderly patients, feeding tube, oral care, pneumonia, psychiatric disorders

## Abstract

We investigated the effect of oral care on the prevention of pneumonia using a clinical scoring scale in elderly patients with psychiatric disorders after the withdrawal of nasogastric feeding tubes. Notably, oral care was effective in preventing pneumonia relapse in these patients.

## Introduction

Nasogastric tube (NGT) feeding is temporarily effective for nutritional management in patients with inadequate oral intake. However, nasogastric intubation has also been implicated as a potential source of infection. In addition, there has been no evidence that tube feeding reduces the risk of pneumonia or pneumonia‐related mortalities 1, 2, 3. Therefore, NGT may be withdrawn after oral hygiene training when results of common tests such as videofluoroscopic examination of swallowing, videoendoscopic evaluation of swallowing, repetitive saliva swallowing test 4, and modified water swallowing test 5 are acceptable. However, some elderly patients with psychiatric disorders cannot be adequately evaluated by these tests owing to psychiatric symptoms and cognitive decline. These factors lead to prolonged use of NGT feeding and pneumonia relapse. Therefore, we investigated the effect of oral care on the prevention of pneumonia in elderly patients with psychiatric disorders.

## Case Reports

We conducted a 12‐month, prospective clinical study. Three consecutive elderly patients with psychiatric disorders, who had recovered from recurrent pneumonia and stopped NGT feeding, were enrolled. All three patients met the criteria of the International Classification of Diseases, 10th revision for their respective disorders 6. The inclusion criteria were as follows: (1) easy communication (e.g., open your mouth, shake your hands) was possible; (2) inability to eat at least one meal each day, but can masticate and swallow a spoonful of soft food, such as jelly; and (3) history of recurrent pneumonia. The time of withdrawal of nasogastric feeding tubes was defined as diagnosis by two physicians.

### Case 1

A 69‐year‐old man with alcoholic dementia had Wernicke's encephalopathy and cerebral infarction at the age of 64 years, and he was started on a daily dose of 1 mg risperidone and 25 mg chlorpromazine due to severe behavioral and psychological symptoms of dementia (e.g., screaming and violence). Starting at the age of 66 years, these symptoms worsened and became more frequent. The daily dose of chlorpromazine was increased to 65 mg, whereas that of risperidone was maintained at 1 mg. Subsequent attempts to reduce his medication dose failed due to degeneration of psychiatric symptoms. From the age of 68 years, he had frequent recurrent pneumonia. His Mini‐Mental Status Examination 7 score was 11.

### Case 2

A 75‐year‐old man with mental retardation with The Wechsler Adult Intelligence Scale‐Revised (WAIS‐R) 8 score of 45 frequently experienced worsen psychiatric symptoms (e.g., screaming, refusing care), appetite loss, and pneumonia from the age of 73 years. However, medications were not prescribed to attenuate his loss of activity of daily living due to side effects of psychotropic drugs. Because he had been bedridden for an extended period, the muscular strength of his legs declined and he used a wheelchair.

### Case 3

An 87‐year‐old woman with mental retardation and WAIS‐R 8 score 40 had been prescribed a daily dose of 10 mg olanzapine due to screaming and violence at the age of 81 years. Subsequent attempts to reduce her medication dose failed due to degeneration of psychiatric symptoms. At the age of 83 years, she sustained bilateral femoral fracture. Gait rehabilitation was offered, but she refused it and chose to use a wheelchair. Starting at the age of 85 years, she had frequent urinary tract infections and pneumonia.

In all patients, the activities of daily living were gradually reduced (including worsening of psychiatric symptoms, relapse of pneumonia, and incidence of complications) and dysphagia worsened, resulting in repeated need for NGT insertion and use of intravenous antibiotic infusions for recurring pneumonia. To address these problems, an oral hygiene training was initiated. This included thrice daily massaging of the parotid, submandibular, and sublingual glands and brushing of the soft palate and the back of the tongue using swabs, for about 10 min at a time. The nurses and caregivers were educated on these methods. The oral hygiene of patients was evaluated using a scale developed at Yuzuriha Hospital, which was based on the Japanese Society of Dysphagia Rehabilitation 9, for the prevention of pneumonia. This scale assesses sitting in an upright posture, palsy, pronunciation, lip protrusion, tongue movement, pharyngeal reflex, and halitosis. Scores for each item range from 0 to 3, with higher scores indicating increased swallowing function normality.

Patients were examined for signs of pneumonia relapse, serum albumin levels, and the presence of bedsores at baseline immediately after withdrawal of the NGT and then once every month during the 12‐month study period. Pneumonia was defined as (1) the development of new progressive infiltrates on chest radiograph, (2) C‐reactive protein >0.3 mg/dL, and (3) pneumonia diagnosis by two physicians. The assessment and training were conducted by one geriatric psychiatrist and three dental professionals. This study was approved by the Yuzuriha Hospital ethics committee.

The patients’ scores on the oral hygiene scale are presented in Table 1. During the follow‐up period, the swallowing function of all patients remained stable or improved. There were no other abnormalities, such as signs of pneumonia relapse, abnormal serum albumin levels, or bedsores in any of the patients. Need for NGT reinsertion was not observed in any patient.

**Table 1:** The findings from three elderly patients with psychiatric disorders before and after oral hygiene training using a scale developed at the Yuzuriha Hospital.^a^

	Base line^b^	At 12 months
Sitting in an upright posture	1.0	3.0
Palsy	3.0	3.0
Pronunciation	1.67	3.0
Lip protrusion	1.0	2.67
Tongue movement	0.33	1.33
Pharyngeal reflex	3.0	3.0
Halitosis	3.0	3.0

a Severe dysfunction = 0, moderate dysfunction =1, mild dysfunction = 2, normal = 3.

b Base line: immediately after withdrawal of the nasogastric tube.

## Discussion

As age increases, there are changes in gastrointestinal flora due to decreased gastric acid secretion, increased aspiration, loss of appetite, reduced salivation, and increased use of dentures, which lead to pneumonia 10, 11. Because patients with pneumonia have a low nutritional state, NGT feeding is used for temporary nutritional management. However, tube feeding is also associated with a higher incidence of pneumonia 12.

Almost all of staff members in hospitals and nursing homes understand that appropriate oral hygiene can prevent pneumonia, thus avoiding NGT reinsertion 13. However, some elderly patients with psychiatric disorders, including schizophrenia, mental retardation, and dementia, often poorly react when receiving assistance and examinations due to psychiatric symptoms and cognitive decline 14, 15. In a previous report, only 30% nurses received oral hygiene training 12. In this study, oral care in elderly patients with psychiatric disorders was investigated to prevent pneumonia and NGT reinsertion. The three focal points were as follows: (1) To assess the patients’ activities of daily living using the oral hygiene scale. This scale mostly contains the observations of the patients by staff without video endoscopy and contrast drugs. These observations are very important because some elderly patients with psychiatric disorders are more difficult to follow and often cannot understand the instructions necessary for examinations, such as “frequently swallow your saliva for thirty‐seconds.” (2) Oral hygiene training. This training strengthens the masseter, orbicularis oris, and tongue muscles. (3) Education of staff. The nurses and caregivers were educated with regard to the methods of assessment and training by the Nutrition Dysphagia Rehabilitation Committee of Yuzuriha Hospital once a month. Consequently, we were successful in preventing pneumonia and NGT reinsertion for 1 year for the three elderly patients with psychiatric disorders.

## Conclusion

Oral hygiene assessment and training for nurses and caregivers were effective in preventing pneumonia relapse and managing nutritional intake in these three elderly patients with psychiatric disorders.

## Authorship

AH: collected and analyzed data, designed the study and wrote the manuscript. AH, MH,and RT: followed up the patients. RT: supervised the whole study process. All authors read and approved the final manuscript.

## Conflict of Interest

None declared.
